# 2446 Feasibility, acceptability, and appropriateness of the menstrual cup for short-term non-surgical management of vesicovaginal fistula (VVF) among potential users and stakeholders

**DOI:** 10.1017/cts.2018.74

**Published:** 2018-11-21

**Authors:** Nessa E. Ryan

**Affiliations:** H+H Clinical and Translational Science Institute, NYU

## Abstract

OBJECTIVES/SPECIFIC AIMS: To examine how women with OF in Ghana develop strategies for coping in the absence of access to successful surgical repair. To assess the feasibility, acceptability, and appropriateness of an innovation to support coping among women with OF seeking care in a health facility in Ghana. To examine the perceived facilitators and barriers to implementation among additional OF stakeholders regarding the innovation. METHODS/STUDY POPULATION: This study uses a sequential exploratory mixed methods design. The population of study is women in Ghana living with obstetric fistula, as well as additional fistula stakeholders (programmers, policy makers, community leaders). To get an understanding of usual leakage, women carried out at baseline a pad test, where they wore a sanitary pad for 2 hours and leaked freely. We subtracted the dry pad weight from the wet pad weight to estimate urine leakage in mL. Then women inserted the cup for 2 hours and again wore a pad and urine leakage was estimated. Acceptability among women with vesicovaginal fistula was measured by questionnaire. Acceptability among additional stakeholders was examined by semistructured interview. Appropriateness was assessed among the user, additional stakeholders, and organizational setting. RESULTS/ANTICIPATED RESULTS: We observed a 61% mean reduction in leakage with the cup which was also perceived by cup users as a reduction in wetness. Notably, one participant who had 4 previous surgical attempts, experienced a 78% reduction in leakage. No adverse events attributable to use of the cup were observed, unlike some of the strategies women currently use to manage leakage. Acceptability was high as most women could easily insert, remove, and wear the cup over the 2-hour period and fistula stakeholders indicated the innovation content and complexity were acceptable. In community interviews, women shared various coping and self-care strategies to manage their leaking, other related impairments, and stigma. Women using the cup in the health facility expressed that it was useful. Additional stakeholders found the cup a low-cost, low-tech solution to supplement existing programs. Within the stakeholder interviews we heard that the cultural norms and existing activities of the potential implementation partners align with the innovation approach. Stakeholders revealed various implementation facilitators and barriers. The facilitators to implementation reported in the interviews were related to the intervention and organization characteristics in particular. Stakeholders perceived a relative advantage to self-management. Stakeholders had concerns regarding whether women would find the insertable device acceptable and appropriate—questioning whether potential users would have access to water, soap, and safe space to empty cup. DISCUSSION/SIGNIFICANCE OF IMPACT: The innovation is efficacious, acceptable, adds to current coping strategies, and fits within existing fistula programs. Stakeholders pre-implementation perceptions highlight the importance of partnerships and the need for an evidence base related to effectiveness, acceptability, and cost. Challenges to address include access to resources within these contexts (water, soap, and safe space) and development appropriate counseling message.

